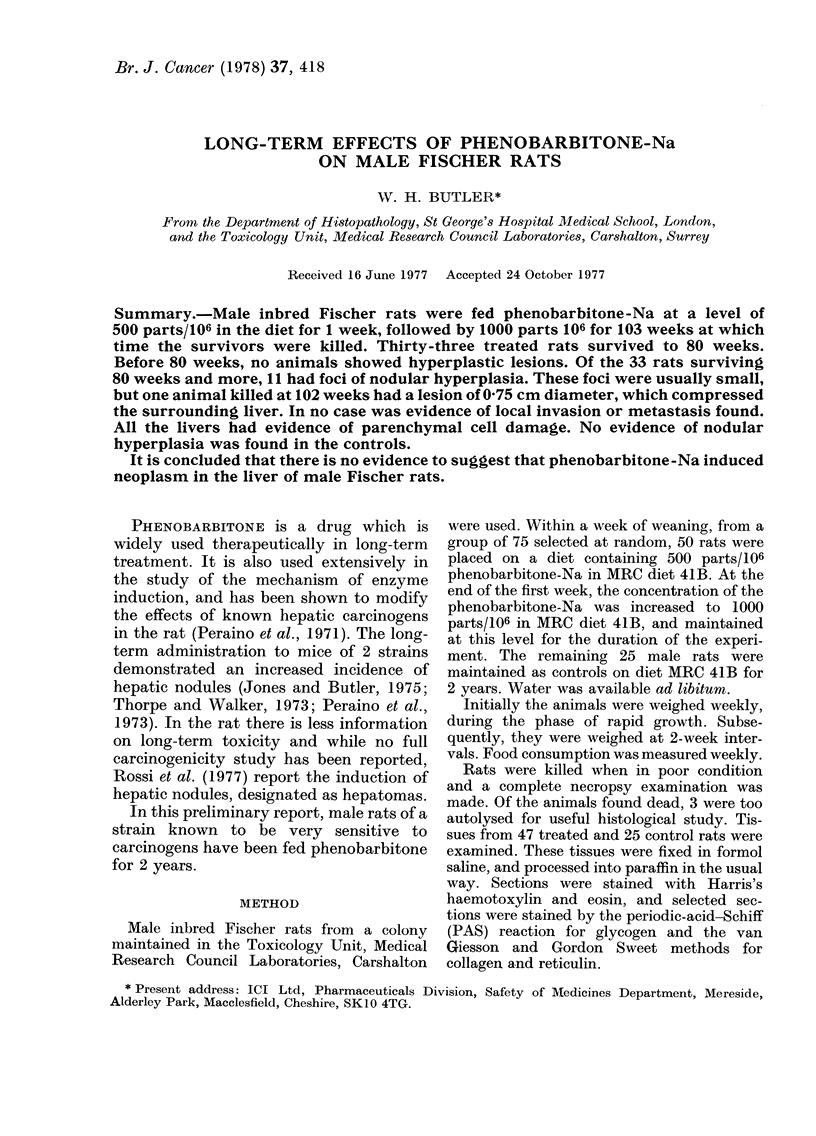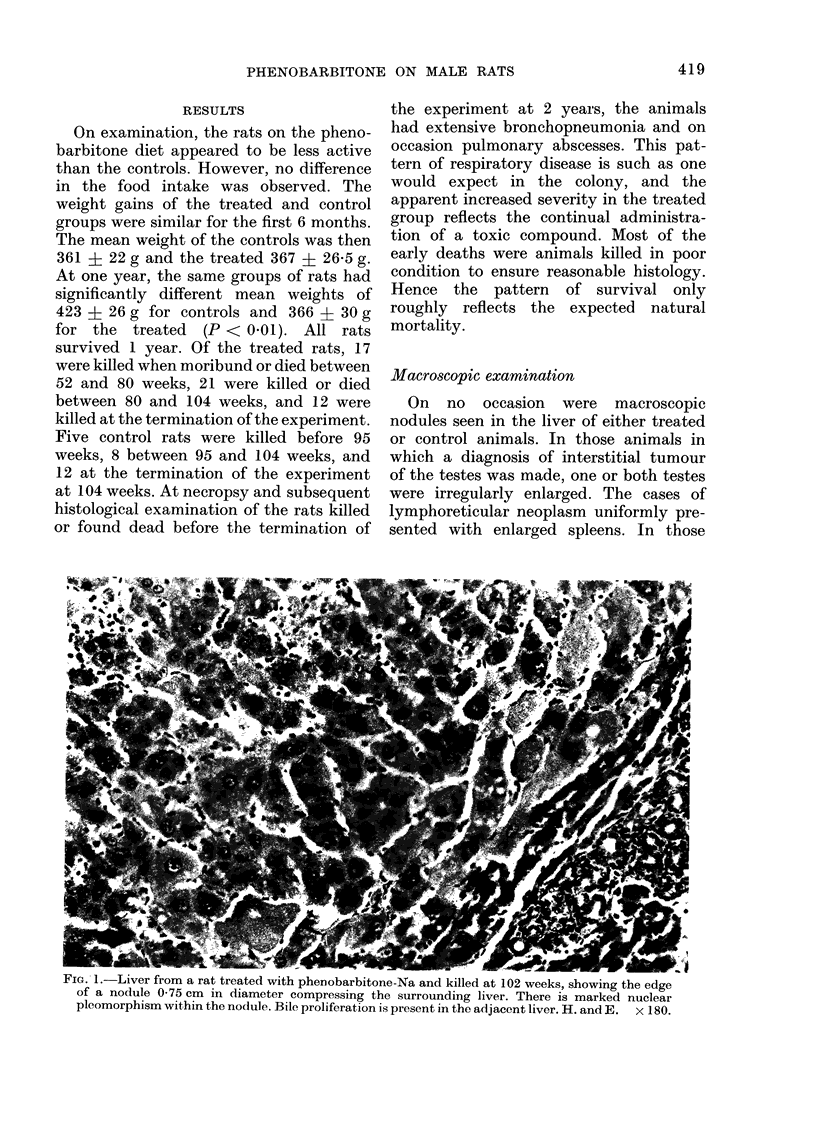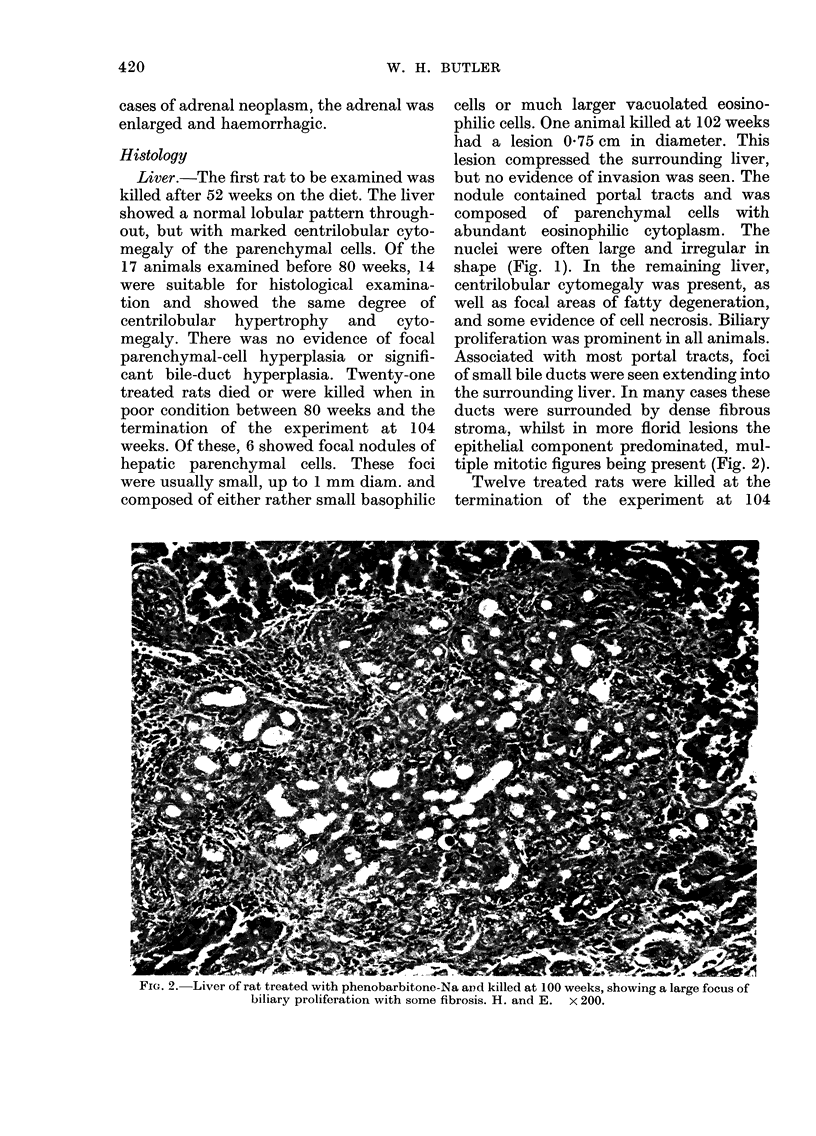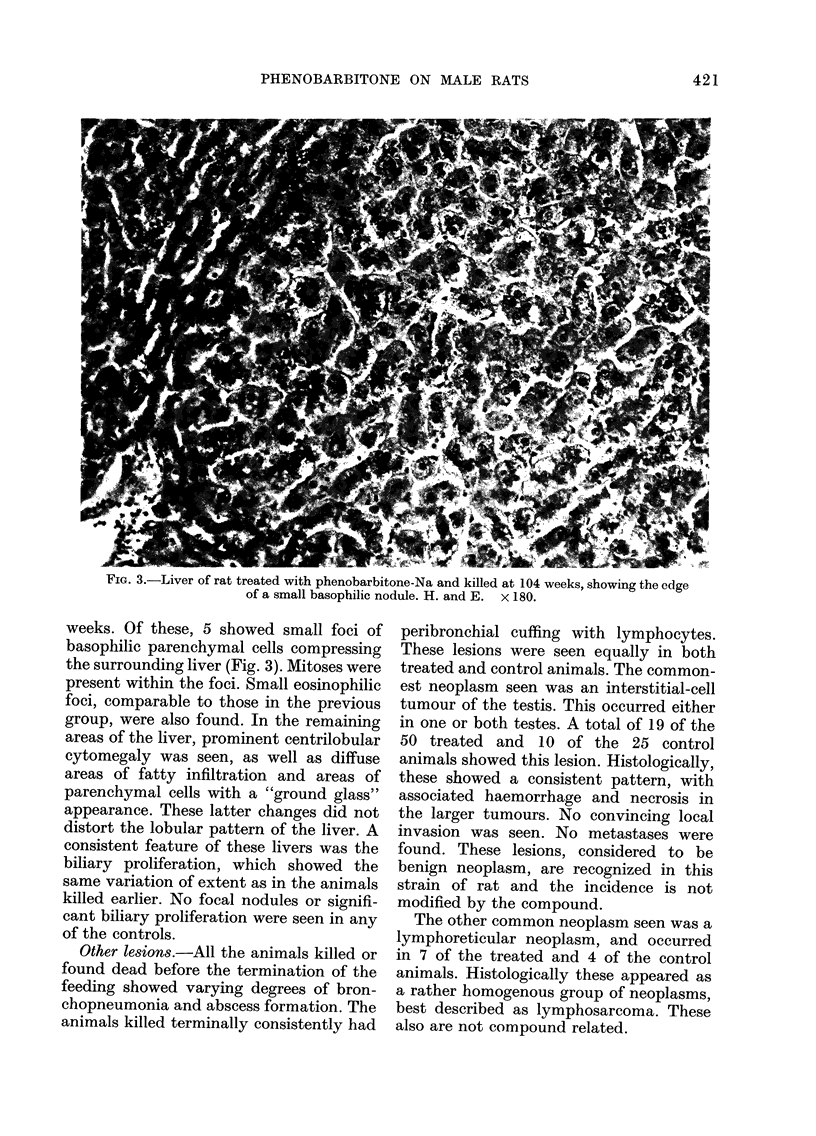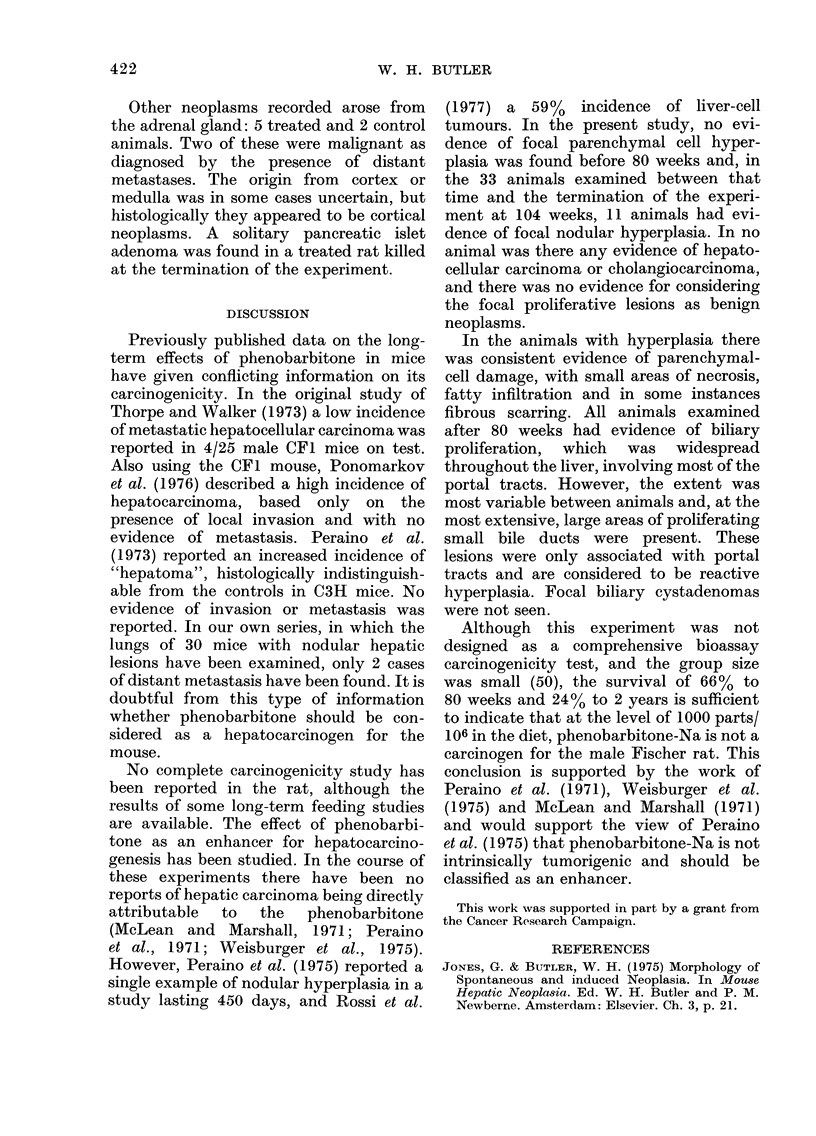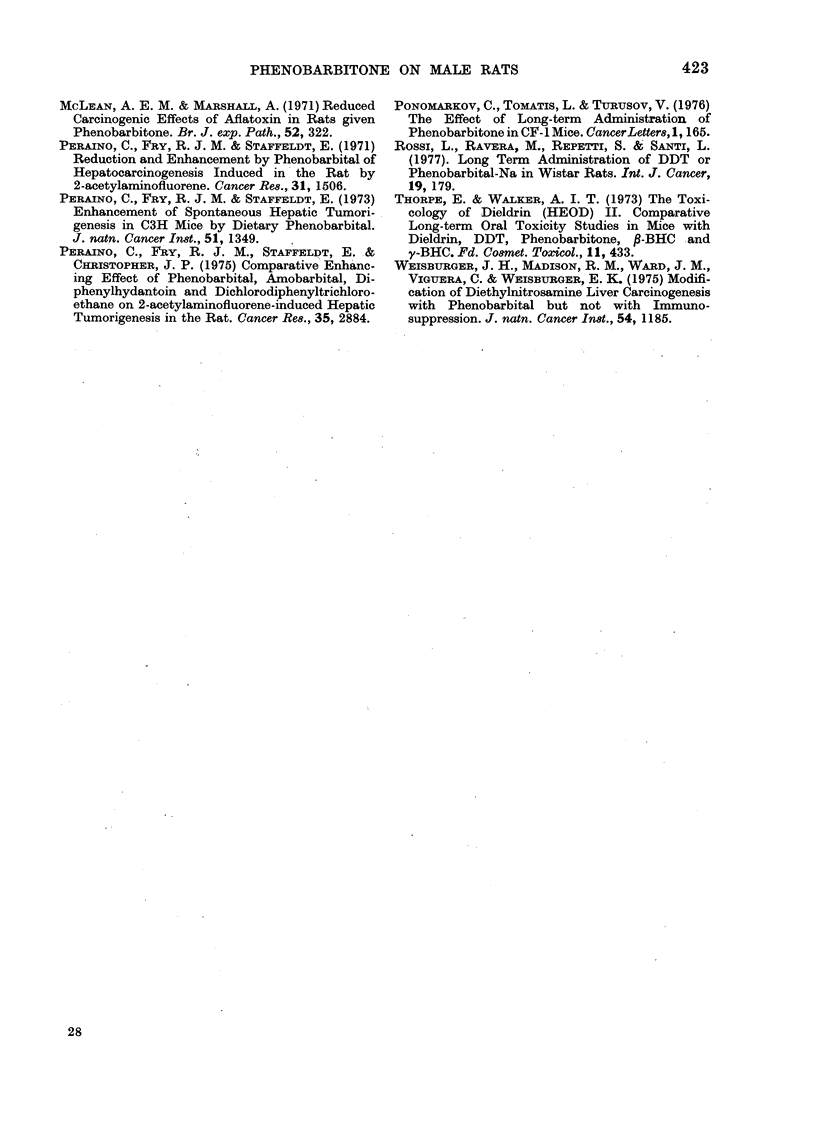# Long-term effects of phenobarbitone-Na on male Fischer rats.

**DOI:** 10.1038/bjc.1978.62

**Published:** 1978-03

**Authors:** W. H. Butler

## Abstract

**Images:**


*Br. J. Cancer* (1978) **37**, 418

### LONG-TERM EFFECTS OF PHENOBARBITONE-Na ON MALE FISCHER RATS

W. H. BUTLER*

*From the Department of Histopathology, St George's Hospital Medical School, London, and the Toxicology Unit, Medical Research Council Laboratories, Carshalton, Surrey*

Received 16 June 1977 Accepted 24 October 1977

**Summary.—Male inbred Fischer rats were fed phenobarbitone-Na at a level of 500 parts/$10^6$ in the diet for 1 week, followed by 1000 parts $10^6$ for 103 weeks at which time the survivors were killed. Thirty-three treated rats survived to 80 weeks. Before 80 weeks, no animals showed hyperplastic lesions. Of the 33 rats surviving 80 weeks and more, 11 had foci of nodular hyperplasia. These foci were usually small, but one animal killed at 102 weeks had a lesion of 0·75 cm diameter, which compressed the surrounding liver. In no case was evidence of local invasion or metastasis found. All the livers had evidence of parenchymal cell damage. No evidence of nodular hyperplasia was found in the controls.**

**It is concluded that there is no evidence to suggest that phenobarbitone-Na induced neoplasm in the liver of male Fischer rats.**

PHENOBARBITONE is a drug which is widely used therapeutically in long-term treatment. It is also used extensively in the study of the mechanism of enzyme induction, and has been shown to modify the effects of known hepatic carcinogens in the rat (Peraino *et al.*, 1971). The long-term administration to mice of 2 strains demonstrated an increased incidence of hepatic nodules (Jones and Butler, 1975; Thorpe and Walker, 1973; Peraino *et al.*, 1973). In the rat there is less information on long-term toxicity and while no full carcinogenicity study has been reported, Rossi *et al.* (1977) report the induction of hepatic nodules, designated as hepatomas.

In this preliminary report, male rats of a strain known to be very sensitive to carcinogens have been fed phenobarbitone for 2 years.

### METHOD

Male inbred Fischer rats from a colony maintained in the Toxicology Unit, Medical Research Council Laboratories, Carshalton were used. Within a week of weaning, from a group of 75 selected at random, 50 rats were placed on a diet containing 500 parts/$10^6$ phenobarbitone-Na in MRC diet 41B. At the end of the first week, the concentration of the phenobarbitone-Na was increased to 1000 parts/$10^6$ in MRC diet 41B, and maintained at this level for the duration of the experiment. The remaining 25 male rats were maintained as controls on diet MRC 41B for 2 years. Water was available *ad libitum.*

Initially the animals were weighed weekly, during the phase of rapid growth. Subsequently, they were weighed at 2-week intervals. Food consumption was measured weekly.

Rats were killed when in poor condition and a complete necropsy examination was made. Of the animals found dead, 3 were too autolysed for useful histological study. Tissues from 47 treated and 25 control rats were examined. These tissues were fixed in formol saline, and processed into paraffin in the usual way. Sections were stained with Harris's haemotoxylin and eosin, and selected sections were stained by the periodic-acid–Schiff (PAS) reaction for glycogen and the van Giesson and Gordon Sweet methods for collagen and reticulin.

* Present address: ICI Ltd, Pharmaceuticals Division, Safety of Medicines Department, Mereside, Alderley Park, Macclesfield, Cheshire, SK10 4TG.

### RESULTS

On examination, the rats on the phenobarbitone diet appeared to be less active than the controls. However, no difference in the food intake was observed. The weight gains of the treated and control groups were similar for the first 6 months. The mean weight of the controls was then $361 \pm 22$ g and the treated $367 \pm 26{\cdot}5$ g. At one year, the same groups of rats had significantly different mean weights of $423 \pm 26$ g for controls and $366 \pm 30$ g for the treated ($P < 0{\cdot}01$). All rats survived 1 year. Of the treated rats, 17 were killed when moribund or died between 52 and 80 weeks, 21 were killed or died between 80 and 104 weeks, and 12 were killed at the termination of the experiment. Five control rats were killed before 95 weeks, 8 between 95 and 104 weeks, and 12 at the termination of the experiment at 104 weeks. At necropsy and subsequent histological examination of the rats killed or found dead before the termination of the experiment at 2 years, the animals had extensive bronchopneumonia and on occasion pulmonary abscesses. This pattern of respiratory disease is such as one would expect in the colony, and the apparent increased severity in the treated group reflects the continual administration of a toxic compound. Most of the early deaths were animals killed in poor condition to ensure reasonable histology. Hence the pattern of survival only roughly reflects the expected natural mortality.

### *Macroscopic examination*

On no occasion were macroscopic nodules seen in the liver of either treated or control animals. In those animals in which a diagnosis of interstitial tumour of the testes was made, one or both testes were irregularly enlarged. The cases of lymphoreticular neoplasm uniformly presented with enlarged spleens. In those

FIG. 1.—Liver from a rat treated with phenobarbitone-Na and killed at 102 weeks, showing the edge of a nodule 0·75 cm in diameter compressing the surrounding liver. There is marked nuclear pleomorphism within the nodule. Bile proliferation is present in the adjacent liver. H. and E. × 180.

cases of adrenal neoplasm, the adrenal was enlarged and haemorrhagic.

### *Histology*

*Liver.*—The first rat to be examined was killed after 52 weeks on the diet. The liver showed a normal lobular pattern throughout, but with marked centrilobular cytomegaly of the parenchymal cells. Of the 17 animals examined before 80 weeks, 14 were suitable for histological examination and showed the same degree of centrilobular hypertrophy and cytomegaly. There was no evidence of focal parenchymal-cell hyperplasia or significant bile-duct hyperplasia. Twenty-one treated rats died or were killed when in poor condition between 80 weeks and the termination of the experiment at 104 weeks. Of these, 6 showed focal nodules of hepatic parenchymal cells. These foci were usually small, up to 1 mm diam. and composed of either rather small basophilic cells or much larger vacuolated eosinophilic cells. One animal killed at 102 weeks had a lesion 0·75 cm in diameter. This lesion compressed the surrounding liver, but no evidence of invasion was seen. The nodule contained portal tracts and was composed of parenchymal cells with abundant eosinophilic cytoplasm. The nuclei were often large and irregular in shape (Fig. 1). In the remaining liver, centrilobular cytomegaly was present, as well as focal areas of fatty degeneration, and some evidence of cell necrosis. Biliary proliferation was prominent in all animals. Associated with most portal tracts, foci of small bile ducts were seen extending into the surrounding liver. In many cases these ducts were surrounded by dense fibrous stroma, whilst in more florid lesions the epithelial component predominated, multiple mitotic figures being present (Fig. 2).

Twelve treated rats were killed at the termination of the experiment at 104

Fig. 2.—Liver of rat treated with phenobarbitone-Na and killed at 100 weeks, showing a large focus of biliary proliferation with some fibrosis. H. and E. ×200.

FIG. 3.—Liver of rat treated with phenobarbitone-Na and killed at 104 weeks, showing the edge of a small basophilic nodule. H. and E. ×180.

weeks. Of these, 5 showed small foci of basophilic parenchymal cells compressing the surrounding liver (Fig. 3). Mitoses were present within the foci. Small eosinophilic foci, comparable to those in the previous group, were also found. In the remaining areas of the liver, prominent centrilobular cytomegaly was seen, as well as diffuse areas of fatty infiltration and areas of parenchymal cells with a "ground glass" appearance. These latter changes did not distort the lobular pattern of the liver. A consistent feature of these livers was the biliary proliferation, which showed the same variation of extent as in the animals killed earlier. No focal nodules or significant biliary proliferation were seen in any of the controls.

*Other lesions.*—All the animals killed or found dead before the termination of the feeding showed varying degrees of bronchopneumonia and abscess formation. The animals killed terminally consistently had peribronchial cuffing with lymphocytes. These lesions were seen equally in both treated and control animals. The commonest neoplasm seen was an interstitial-cell tumour of the testis. This occurred either in one or both testes. A total of 19 of the 50 treated and 10 of the 25 control animals showed this lesion. Histologically, these showed a consistent pattern, with associated haemorrhage and necrosis in the larger tumours. No convincing local invasion was seen. No metastases were found. These lesions, considered to be benign neoplasm, are recognized in this strain of rat and the incidence is not modified by the compound.

The other common neoplasm seen was a lymphoreticular neoplasm, and occurred in 7 of the treated and 4 of the control animals. Histologically these appeared as a rather homogenous group of neoplasms, best described as lymphosarcoma. These also are not compound related.

Other neoplasms recorded arose from the adrenal gland: 5 treated and 2 control animals. Two of these were malignant as diagnosed by the presence of distant metastases. The origin from cortex or medulla was in some cases uncertain, but histologically they appeared to be cortical neoplasms. A solitary pancreatic islet adenoma was found in a treated rat killed at the termination of the experiment.

### DISCUSSION

Previously published data on the long-term effects of phenobarbitone in mice have given conflicting information on its carcinogenicity. In the original study of Thorpe and Walker (1973) a low incidence of metastatic hepatocellular carcinoma was reported in 4/25 male CF1 mice on test. Also using the CF1 mouse, Ponomarkov *et al.* (1976) described a high incidence of hepatocarcinoma, based only on the presence of local invasion and with no evidence of metastasis. Peraino *et al.* (1973) reported an increased incidence of "hepatoma", histologically indistinguishable from the controls in C3H mice. No evidence of invasion or metastasis was reported. In our own series, in which the lungs of 30 mice with nodular hepatic lesions have been examined, only 2 cases of distant metastasis have been found. It is doubtful from this type of information whether phenobarbitone should be considered as a hepatocarcinogen for the mouse.

No complete carcinogenicity study has been reported in the rat, although the results of some long-term feeding studies are available. The effect of phenobarbitone as an enhancer for hepatocarcinogenesis has been studied. In the course of these experiments there have been no reports of hepatic carcinoma being directly attributable to the phenobarbitone (McLean and Marshall, 1971; Peraino *et al.*, 1971; Weisburger *et al.*, 1975). However, Peraino *et al.* (1975) reported a single example of nodular hyperplasia in a study lasting 450 days, and Rossi *et al.* (1977) a 59% incidence of liver-cell tumours. In the present study, no evidence of focal parenchymal cell hyperplasia was found before 80 weeks and, in the 33 animals examined between that time and the termination of the experiment at 104 weeks, 11 animals had evidence of focal nodular hyperplasia. In no animal was there any evidence of hepatocellular carcinoma or cholangiocarcinoma, and there was no evidence for considering the focal proliferative lesions as benign neoplasms.

In the animals with hyperplasia there was consistent evidence of parenchymal-cell damage, with small areas of necrosis, fatty infiltration and in some instances fibrous scarring. All animals examined after 80 weeks had evidence of biliary proliferation, which was widespread throughout the liver, involving most of the portal tracts. However, the extent was most variable between animals and, at the most extensive, large areas of proliferating small bile ducts were present. These lesions were only associated with portal tracts and are considered to be reactive hyperplasia. Focal biliary cystadenomas were not seen.

Although this experiment was not designed as a comprehensive bioassay carcinogenicity test, and the group size was small (50), the survival of 66% to 80 weeks and 24% to 2 years is sufficient to indicate that at the level of 1000 parts/$10^6$ in the diet, phenobarbitone-Na is not a carcinogen for the male Fischer rat. This conclusion is supported by the work of Peraino *et al.* (1971), Weisburger *et al.* (1975) and McLean and Marshall (1971) and would support the view of Peraino *et al.* (1975) that phenobarbitone-Na is not intrinsically tumorigenic and should be classified as an enhancer.

This work was supported in part by a grant from the Cancer Research Campaign.

### REFERENCES

Jones, G. & Butler, W. H. (1975) Morphology of Spontaneous and induced Neoplasia. In *Mouse Hepatic Neoplasia.* Ed. W. H. Butler and P. M. Newberne. Amsterdam: Elsevier. Ch. 3, p. 21.

McLean, A. E. M. & Marshall, A. (1971) Reduced Carcinogenic Effects of Aflatoxin in Rats given Phenobarbitone. *Br. J. exp. Path.*, **52**, 322.

Peraino, C., Fry, R. J. M. & Staffeldt, E. (1971) Reduction and Enhancement by Phenobarbital of Hepatocarcinogenesis Induced in the Rat by 2-acetylaminofluorene. *Cancer Res.*, **31**, 1506.

Peraino, C., Fry, R. J. M. & Staffeldt, E. (1973) Enhancement of Spontaneous Hepatic Tumorigenesis in C3H Mice by Dietary Phenobarbital. *J. natn. Cancer Inst.*, **51**, 1349.

Peraino, C., Fry, R. J. M., Staffeldt, E. & Christopher, J. P. (1975) Comparative Enhancing Effect of Phenobarbital, Amobarbital, Diphenylhydantoin and Dichlorodiphenyltrichloroethane on 2-acetylaminofluorene-induced Hepatic Tumorigenesis in the Rat. *Cancer Res.*, **35**, 2884.

Ponomarkov, C., Tomatis, L. & Turusov, V. (1976) The Effect of Long-term Administration of Phenobarbitone in CF-1 Mice. *Cancer Letters*, **1**, 165.

Rossi, L., Ravera, M., Repetti, S. & Santi, L. (1977). Long Term Administration of DDT or Phenobarbital-Na in Wistar Rats. *Int. J. Cancer*, **19**, 179.

Thorpe, E. & Walker, A. I. T. (1973) The Toxicology of Dieldrin (HEOD) II. Comparative Long-term Oral Toxicity Studies in Mice with Dieldrin, DDT, Phenobarbitone, $\beta$-BHC and $\gamma$-BHC. *Fd. Cosmet. Toxicol.*, **11**, 433.

Weisburger, J. H., Madison, R. M., Ward, J. M., Viguera, C. & Weisburger, E. K. (1975) Modification of Diethylnitrosamine Liver Carcinogenesis with Phenobarbital but not with Immunosuppression. *J. natn. Cancer Inst.*, **54**, 1185.